# Prevalence of metabolic syndrome among pregnant women: a systematic review and meta-analysis

**DOI:** 10.1007/s12020-025-04160-8

**Published:** 2025-01-22

**Authors:** A. Mohebi, MM Pathirana, A. Khoja, MR Wittwer, K. Lowe, D. Fisher, S. Kharwadkar, C. Gomes, T. Gamage, E. Toyer, S. Young, MA Arstall, PH Andraweera

**Affiliations:** 1 Adelaide Medical School, The University of Adelaide, Adelaide, SA Australia; 2 Robinson Research Institute, The University of Adelaide, Adelaide, SA Australia; 3 Department of Cardiology, Lyell McEwin Hospital, Elizabeth Vale, Adelaide, SA Australia; 4 Department of Medicine, The Aga Khan University, Karachi, Pakistan

**Keywords:** Pregnancy, Metabolic syndrome, Meta analysis, Obesity, Prevalence, Cardiovascular

## Abstract

**Purpose:**

Metabolic syndrome (MetS) is a cluster of risk factors that increase the risk of cardiometabolic diseases. The prevalence of MetS and individual components across pregnancy has not been reviewed in the literature. This research was conducted to identify the prevalence of MetS and its components among pregnant women.

**Methods:**

The PubMed, EMBASE, CINAHL, Web of Science and Scopus databases were searched. The review protocol is registered in PROSPERO (CRD42023460729). Quality assessment was performed using the JBI critical appraisal checklist. The study selection, data extraction and data analyses were performed in accordance with the MOOSE guidelines.

**Results:**

The prevalence of MetS among pregnant women was 16.3%, (n = 3946). The prevalences for individual MetS components were: low HDL, 12.3% (n = 1108); high fasting glucose, 16.2% (n = 2333); high triglycerides, 48.5% (n = 2880); obesity, 42.7% (n = 5162) and high blood pressure 37.7% (n = 828). According to the definitions used to diagnose MetS, the prevalences were 18.2% according to the World Health Organization, 15.0% according to the International Diabetes Federation and 17.2% according to the National Cholesterol Education Program Adult Treatment Panel III. When stratified by gestational age at assessment, the prevalence of MetS was 9.9% before 16 weeks’ and 24.1% after 20 weeks’ of gestation.

**Conclusion:**

This review demonstrates that MetS is detected in approximately one-fifth of pregnant women. Screening for MetS and its components during pregnancy may help identify young women at risk for future cardiovascular disease.

## Introduction

Cardiovascular disease (CVD) is the world’s leading cause of death, accounting for 17.9 million deaths globally each year [1]. In 2021, Ischaemic heart disease was identified as the leading cause of CVD [2]. Despite significant advancements in preventive health, modifiable risk factors continue to contribute to the major burden of ischaemic heart disease-related CVD. According to the Global Burden of Disease study which included data from 204 countries, high blood pressure, elevated LDL cholesterol, high fasting blood glucose and obesity attributed to 10.8 million, 3.8 million, 2.3 million and 1.9 million cardiovascular deaths, respectively [2].

Metabolic syndrome (MetS) is a multifaceted risk factor for CVD, characterised by a cluster of modifiable cardio-metabolic factors, including abdominal obesity and insulin resistance [3]. A meta-analysis of 87 studies that included 951,083 participants found that MetS increased the risk of CVD with a relative risk of 2.35 (95% CI, 2.02–2.73) [3, 4]. Additionally, MetS was associated with significantly higher risks of all-cause mortality, CVD mortality, myocardial infarction and stroke, with risk ratios ranging from 1.6 to 2.9, even in individuals without diabetes [2].

The prevalence of MetS is increasing worldwide, with an estimated global prevalence ranging from 12.5–31.4% among adults, depending on which definition is used [5]. The most common criteria used to diagnose MetS are The National Cholesterol Education Program Adult Treatment Panel III (NCEP-ATP III), International Diabetes Federation (IDF) and World Health Organisation (WHO) guidelines [6–8]. These criteria use combinations of the following factors to define MetS: BMI, triglyceride levels, HDL levels, blood pressure and fasting glucose as shown below [3].

**Table:** 

Risk factors	IDF (2005)	NCEP-ATP III (2001)	WHO (2008)
Absolutely required	Waist circumference ≥80 cm (F), ≥94 (M)	None	Insulin resistance or other diabetes
Criteria	Obesity plus two additional criteria	Any three of the criteria	Insulin resistance or diabetes plus two the additional criteria
Central obesity	Already required	Waist circumference: ≥88 cm (F), ≥102 cm (M)	Female BMI > 30 kg/m^2^
Raised fasting glucose	≥100 mg/dL	≥100 mg/dL	Already required
Raised triglycerides	≥150 mg/dL	≥150 mg/dL	≥150 mg/dL
Reduced HDL cholesterol	<50 mg/dL (F), <40 mg/dL (M)	<50 mg/dL (F), <40 mg/dL (M)	
Raised blood pressure	SBP > 130 mm Hg orDBP >85 mm Hg	SBP > 130 mmHg or DBP > 85 mmHg	SBP/DBP ≥ 140/90 mmHg

Despite above guidelines, there are no established healthy metabolic and vascular criteria that are specific for pregnancy. Therefore, MetS in pregnancy is diagnosed using the standard definitions or population specific criteria, which may lead to an overestimation of its prevalence. In addition to the rising rates of MetS among adults, a recent systematic review conducted in the year 2020 on modelling analysis, estimated a global prevalence of MetS at 2.8% for children and 4.8% for adolescents [5]. These findings suggest that approximately 35.5 million young people are living with MetS globally [5].

Pregnancy is considered a cardiovascular stress test for women, as the cardiovascular system undergoes significant structural and haemodynamic changes to meet the requirements of the growing foetus [9]. A maternal inability to adapt to these physiological changes can increase the risk of developing major pregnancy complications and reveal previous silent cardiometabolic pathologies. Therefore, detecting MetS during pregnancy provides an opportunity to engage in early intervention strategies to prevent future CVD [9].

This systematic review and meta-analysis aims to identify the prevalence of MetS and its components among pregnant women. Additionally, we assessed the prevalence of MetS in pregnancy, stratified by the diagnostic criteria used and the gestational age at assessment.

## Methods

### Data sources and search strategy

We conducted a systematic review and meta-analysis in compliance with the Meta-analysis of Observational Studies in Epidemiology (MOOSE) guidelines (Supplemetary file 1) [10]. The review protocol was registered in PROSPERO (CRD42023460729). Electronic databases including PubMed, EMBASE, CINAHL, Web of Science and Scopus were systematically searched until 18 August 2023, to identify all published studies that reported the prevalence of MetS and its components among pregnant women. Subsequently, we did another search from 19 August 2023 until 28 May 2024, to identify potential studies that reported on prevalence of MetS and its components among pregnant women.

**Fig. 1:**
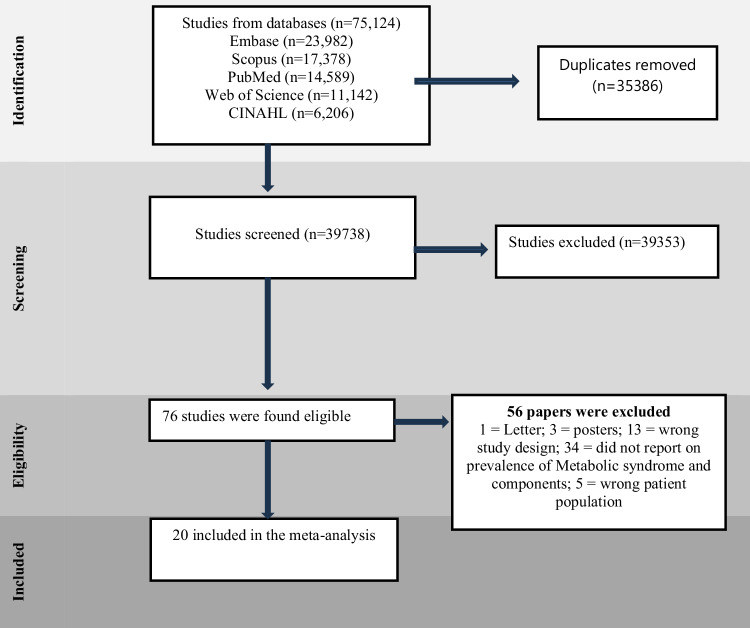
Study selection process

The combination of search terms and medical subject heading (MeSH) terms used are presented in the Supplementary Appendix. All potential eligible studies were included for review, and additional relevant publications were retrieved from the bibliographies of pre-selected primary articles and other sources to identify further potential relevant studies (Fig. 1).

### Study selection

Studies that reported on the prevalence of MetS and its components during pregnancy were included. As there are many guidelines used to define MetS, including the WHO, IDF and NCEP-ATP III criteria, studies that used any definition were eligible for inclusion (Table 1). Studies that assessed MetS at any gestion during pregnancy were also eligible. Studies published in English, undertaken in humans and published in peer-reviewed journals were selected for inclusion. Studies that reported on a mixed population of pregnant and non-pregnant women were excluded. The title and abstract of each article were reviewed by one primary (AM and PA) and one secondary reviewer (MP, AK, MW, KL, DJ, SK, CG, TG, ET, SY) and studies that met the eligibility criteria were retrieved for evaluation of the full text.

**Table 1:** Characteristics of the included studies

Author Study design Country	Sample size Age group	Parity Time of assessment	Criteria used to diagnose metabolic syndrome	Inclusion criteria	Exclusion criteria	Prevalence of Mets	Prevalence of Mets components
Bjelanović et al. [13] Cross-sectional, prospective study Bosnia or Croatia	180 Not specified but between 18–40	Not specified First weeks of pregnancy	WHO	Women hospitalised in the third trimester of pregnancy, no later than 48 h after delivery.	Women <18 years of age, in the first or second trimester of pregnancy, who had doctor confirmed mental illness or mental disorder before pregnancy, mental retardation, with a diagnosed foetal anomaly, and those with severe complications during pregnancy and delivery (eclampsia, placental abruption, increased bleeding during pregnancy and / or childbirth) were excluded.	Assuming that Table 1 is being reported as n (%) and that MSY is MetS (no definition given) BMI > 30 = 2 (3.3%), MSY = 60 (100%), Control = 0 (0%) = 34.4% (n = 62)	Assuming that Table 1 is being reported as n (%) and that MSY is MetS (no definition given) Hypertriglyceridemia = 87.8% (n = 158) Low HDL = 1.1% (n = 2) Obesity = 66% (n = 119) Hyperglycaemia = 35% (n = 63) Hypertension = 47.2% (n = 85)
Bartha et al. [12] Cohort Study Spain	90 Control = 28 0.5 ± 5.0, Late onset gestational diabetes = 30 0.6 ± 4.2, Gestational hypertensio n = 30.2 ± 4.4, Preeclamps ia = 28.9 ± 5.0	Nulliparous 54% (n = 49) After 20 weeks of pregnancy	WHO/NCEP	Control group: non-diabetic normotensive, age-, gestational age- and parity frequency matched (group matching) pregnant women. Women were followed up until birth and were evaluated at 40 days after delivery. All of them remained normotensive	Women with gestational diabetes diagnosed at early pregnancy (< 14 weeks of gestation) were excluded for considering that a substantial proportion of them could have an undiagnosed pre-pregnancy diabetes mellitus	WHO: late gestational diabetes = 3.3%, gestational hypertension = 35%, PE = 30% NCEP: Late gestational diabetes = 10%, gestational hypertension = 20%, PE = 30%	Fasting hyperglycaemia >105 mg/dL) = 10% Obesity BMI = 24.4%, Hypertriglyceridemi a (>270mg/dL) = 32.2%, Hypo hDL-c (<40mg/dL) = 8.9%.
Bartakova et al. [29] Cross sectional observational Switzerland	455 30-36 years	24-30 weeks	IDF/WHO	Positive screening for GDM by OGTT at mid gestation, singleton pregnancy and Caucasian origin.	Pre-existing type I or II diabetes with established treatment before pregnancy, non-Caucasian, multiple pregnancy.	22.6% (103)	
Dabou et al. [14] Crosssectional study Cameroon	604 16-45 years	Nulliparous = 50.09% (276) Primiparou s = 19.42% (107) Multiparou s = 25.41% (140) Grand multiparous = 5.08% (28) 22.78 ± 9.20 weeks	Metabolic syndrome was diagnosed using the HNLBI/AHA/NCEPATP III definition, modified for pregnant women by Chatzi et al. A participant was recorded as having MS if presenting at least three of the following criteria: Pregestational BMI >30 kg/m2; triglyceride s ≥150 mg/dl; HDL cholesterol	Healthy Cameroonian women attending the Dschang district hospital for antenatal care	Recorded diabetes mellitus, cardiovascular diseases, and mental illnesses Multiple pregnancies	17.88% (n = 108)	Pre-pregnancy obesity = 27.32% (n = 165) (95% CI 23.92%–31.01%) Elevated BP = 11.92% High fasting glucose = 4.97% Hypertriglyceridemi a = 58.28% Low HDL cholesterol = 66.23%
Agbozo et al. [31] Prospective cohort Germany	454 20-24 years	Not specified	Presence of three or more risk factors containing dyslipidaemia, hyperglycaemia, hypertension or adiposity	Attending peri-urban and rural healthcare in ghana	Not specified	2.20% (10)	Triglycerides = 20.98 (95), Total cholesterol = 4.68% (21), Low HDL = 38.31% (20), elevated fasting glucose = 11.03% (50) Hypertension = 2.86% (13) Obesity = 11.75% (53)
Grieger et al. [15] Retrospective cohort Australia	5519 28.9	Nulliparous 14-16 weeks gestation	Internationa l Diabetes Federation (IDF) criteria:	Low risk Nulliparous With singleton pregnancies 14-16 weeks pregnant	High risk of preeclampsia, SGA infants and sPTB because of underlying medical conditions (including known preexisting chronic hypertension on hypertensive medication or having blood pressure > 160/100 mmHg at 15 ± 1 weeks’ gestation), gynaecological history, or three or more miscarriages or terminations of pregnancy or couples who received medical or surgical interventions that could modify pregnancy outcome	684/5519 = 12.4%	Low HDL cholesterol–310/5519 = 5.6% Raised triglycerides–1609/5519 = 29.2% Raised glucose 1948/5519 = 35.3% Raised BP 160/5519 = 2.9% High waist circumference 3356/5519 = 60.8%
Horvath et al. [16] retrospective observational l study Hungary	7373 MetS group: <20 = 7 (3.2%) 20-25 = 20(9.2%) 26-30 = 65 (30%) 31-35 = 82 (36.9%) 36-40 = 34(15.7%) >40 = 11(5.1%) Mean age for MetS group = 30.3 Non MetS group: <20 = 297 (3.9%) 20-25 = 858 (12%) 26-30 = 2424 (33.9%) 31-35 = 2599 (36.3%) 36-40 = 844 (11.8%) >40 = 150 (2.1%) Mean age of the non-MetS group = 29.8	Not specified 12 weeks of gestation	WHO/ NCEP	Pregnancy women suffering from metabolic syndrome in the first trimester of gestation	Not specified	219 (2.9%)	SBP/DBP - 127 ± 37 mmHg/ 82 ± 17, Total cholesterol (4.7–1.4 mmol/L), triglyceride (1.7±mmol/L), HDL (410 + 55 U/L), BMI (23.7–6.7)
Koralegedara et al. [17] Community based cross sectional - comparative study Sri lanka	634 Age not reported	Note reported Less than 12 weeks of gestation	IDF – AHA – WHO	First trimester pregnant women (<12 weeks of gestational age)	Note reported	4.7% (n = 30)	
Lima et al. [18] Cohort study Brasil	200 31.5±8.4 in the MetS group	2.5±2.3 in the MS at 16^th^ week and 1.4±1.6 in the postpartum period 16 weeks gestation and immediate postpartum period	NCEP-ATP-III (National Cholestrol Education Program Adult Treatment Panel III	We included pregnant women confirmed by ultrasound, with gestational age less than or equal to 16 weeks	Pre-gestational diabetes mellitus (DM), suffering from psychiatric disorders, chronic maternal diseases (hypertension, heart disease, kidney disease, epilepsy, kidney failure), congenital malformations, and multiple pregnancies	Prevalence of MetS at 16^th^ weeks of gestation was 3.0% (n = 6) vs 9.7% (n = 18) during the immediate postpartum period.	Abdominal circumference 36% (n = 6), HDL 42% (n = 84), TG 14% (n = 28), fasting blood glucose 0.5% (n = 1), BP 0% (n = 0))
Negrato et al. [19] Cohort Brazil	136 Group IA (28.7 + 5.2) and IB 28.8 + 6.4, Group IIA (31.4 + 5.0) and IIB (33.1 + 5.6)	Singleton 24–28 weeks of gestation	IDF/WHO	Women with singleton pregnancies were assigned to participate if they presented a fasting glycemia. level ≥ 90 mg/dl and/or risk factors for developing GDM.	Not reported	Normoglycemic – 0% (n = 0), mild hyperglycemic – 20% (n = 8), GDM –23.5% (n = 4), and overt GDM – 36.4% (n = 12) in MetS women	Not specified
Rafeeinia et al. [20] Cross sectional Cohort study Gorgan, Iran	50 years	Pregnant women with preeclampsia in third trimester who were referred to Sayyad Shirazi Education al Hospital Gynaecology Department of Golestan University of Medical Sciences	NCEP	PMHx of DM, renal, cardiovascular liver diseases, endocrine disorder, any chronic illnesses	All have preeclampsia	66% (n = 33)	FBS >100 mg/dL (48%) (n = 24), HDL <50 mg/dL (26%, n = 13)), TGL > 150 mg/dL (96% (n = 48)), BMI >30 (16%) (n = 8), SBP >130 mmHg/DBP >85 mmHg (100%) (n = 33).
Schneider et al. [22] prospective cohort, analysis study Adelaide, Australia	1158 25.9 years	Nulliparous 100% 9-16 weeks gestation and (further OGTT at 24-28 weeks to assess for GDM)	IDF	Pregnant nulliparous women recruited for the STOP study at Lyell McEwin hospital, Adelaide between March 2015 and December 2017	Multiparity	9.2% (n = 107)	Of overall population including those with and without MetS: -Waist circumference >80 cm (59.2%) (n = 685), TGL>1.7 mmol/L (13.6%) (n = 157), HDL<1.29mmol/L (16%) (n = 185), SBP >130mmmHg (10.7%) (n = 124), DBP >85mmHg (6.4%) (n = 74), Glucose >5.6mmol/L (8%) (n = 93). BMI (kg/m2)(28.0 mean)
Su et al. [23] RCT Taiwan	112 35.71 Interventio n 35.71 + 4.31 and control 35.82 + 4.28 years	Singleton < 28 weeks of gestation	IDF	(a) singleton pregnancy, (b) less than 28 weeks of gestation, and (c) having at least one of: > 34 years old, prepregnancy BMI ≥ 24 kg/m2, a macrosomia baby [weight ≥ 4.5 kg], history of GDM in a previous pregnancy, and family history of diabetes	Pregnant women with preexisting diabetes (Type 1 or 2), with limited mobility or inability to perform physical exercise, or < 18 years old were excluded	22.32% (n = 25)	(1) Waist circ > = 80 cm – 38%, Fasting glucose (≥ 100 mg/dl) – 7.1%, Systolic blood pressure (≥ 130 mmHg) – 38%, Diastolic blood pressure (≥ 85 mmHg) – 19%, Triglycerides (≥ 150 mg/dl) = 68%, HDL cholesterol (< 50 mg/dl) = 7.1%
Santana et al. [21] Cross Sectional study Cuba	526 20-35 years	Not specified Week 12-14 of pregnancy	NCEP-ATP-III	Classified as normal weight according to BMI, aged 20–35 years, pregnant with a single foetus and at a gestational age of 12–14 weeks (confirmed by US).	Presence of metabolic disease including diabetes mellitus in any of its stages, dyslipidaemia, psychiatric disorders, uterine fibroids and regular consumption of prescription drugs	N = 62 (11.8%)	Not specified
Tavares et al. [24] Crosssectional study Angola	675 24 ± 6.7	Not specified Mean 39.2 ± 1.6	NCPE ATP III, JIS Chatzi et al. Harmonising g the metabolic syndrome, Bartha et al.	Non-diabetic pregnant women receiving care from the public general hospital of Huambo	with established cardiovascular diseases, thyroid dysfunction, excessive alcohol or other drug abuse, current or recent psychiatric treatment (up to 4 months) during pregnancy and caesarean section were excluded	NCEP - 29.2% (n = 197), JIS - 36.6% (n = 247), NCEPT- ATP III Bartha et al. - 1.8% (n = 12) NHLBI/AHA Chatzi et al. - 12.6% (n = 85).	NCEP ATP III, JIS, Bartha, Chatzi NCEP ATP III: Waist circumference: 73.6% (n = 497) Hyperrtriglycerdemi a: 43.4% (n = 293) Decreased HDL: 7.8% (n = 53) Hypertension: 54.8% (n = 370) JIS criteria: Waist circumference: 97.3% (n = 657) Hyperrtriglycerdemi a: 43.4% (n = 293) Decreased HDL: 7.9% (n = 53) Hypertension: 54.8% (n = 370) NCEPT- ATP III Bartha et al.
Trisovic et al. [25] Prospective cohort Serbia	135 32.16 (ranged 20-45) years	MS group only, n = 42: Primip = 2.3%, multip = 97.7% After 24 weeks Second and third trimester	NCEP	monofetal, vital pregnancies with gestational age after 24	Nil	31.1% (n = 42)	Not reported
Sundaram et al. [30] Retrospective cohort USA	27,955	Not specified	NCEP-ATP III	Pregnant women	Not specified	MetS alone 5.75% (1608)	Not specified
Wu et al. [28] China	2527	24-28 weeks	CMA	Singleton pregnancy, adequate language expression and comprehension and informed consent for study participation	Prior diagnosis of diabetes mellites, polycystic ovary syndrome, hypertension, thyroid diseases, acute and chronic infectiouse disease, with evident signs of infection at the time of study or other major disease.	21.65% (522)	
Xia et al. [26] Prospective cohort China	320 27.1±4.0	88 (27.5%) multiparas and 232 (72.5%) primiparas Any time after 12+/- 1 week until delivery	IDF	Pregnant women admitted to the Wuxi People’s Hospital between July 2017 and October 2018	pregnant women with multifetal pregnancy, pre-existing diabetes mellitus, pre-existing hypertension, high risk for preeclampsia, multiple terminations or miscarriages, already known foetal anomaly or abnormal karyotype, increased risk for depression, disability, malignant diseases, severe malnutrition, recent surgery or invasive procedure, and taking supplements or drugs which may modify the results of this study	Elevated BP = 9.7% (N = 31), elevated blood glucose (N = 37) = 11.6%, elevated TG = 10.9% (35), Elevated HDL-c = 7.8% (N = 25)	12.5% (n=40)
Yang et al. [27] Prospective cohort China	765 Aged > = 35: 37.20 ± 2.0 8 Aged 2034: 29.94 ± 2.5 8	Aged > = 35: 3±2.4 Aged 2034: 1±1.2 24-28 weeks	CMA	Singleton	Previous diagnoses of diabetes, hypertension, dyslipidemia, psychiatric disorders, chronic maternal diseases (kidney disease, heart disease, epilepsy, renal failure, etc.), congenital malformations, and multiple pregnancies.	6.7% (n = 51)	BMI > = 25 = 18.6%, abnormal lipid metabolism = 32.5%, hyperlipemia = 7.1%

### Data extraction

Data extraction was independently performed by two reviewers (AM, MW, MP). For each study, data on study design, the definition of MetS used, sample size, mean age of participants, parity, prevalence of MetS and its components (high waist circumference or high BMI, elevated blood pressure, high total cholesterol, high triglycerides, low HDL, high fasting blood glucose) were extracted (detailed in Supp. Table 1).

### Quality assessment

Two reviewers (AM, AK) used the JBI Sumari critical appraisal checklist tool to assess the methodological quality of all individual studies [CHECKLIST FOR PREVALENCE STUDIES Critical Appraisal tools for use in JBI Systematic Reviews]. This checklist comprises nine questions for critically appraising the studies. The studies were scored on a scale of one to ten for low, moderate and high. Of the studies included in the meta-analysis, 11 were of moderate quality and nine were of high quality.

### Data synthesis and analyses

The results were extracted as presented in the published papers, including sample size and the reported number of women with MetS. JBI SUMARI was used to perform the meta-analyses [11]. Subgroup meta-analyses were performed based on the definitions used to diagnose MetS (WHO, IDF and NCEP-ATP III) and the gestational age at the time of diagnosis of MetS (<16 weeks’ gestation, >20 weeks’ gestation). The pooled prevalence was calculated for each outcome measure by applying the random-effects model. The random-effects model was selected as it takes inherent study-to-study variability into account. Substantial heterogeneity among studies was considered if the value of I^2^ statistic was above 50% and χ^2^ p value <0.1.

## Results

A total of 70,427 studies were identified through electronic searches, of which 33,559 were duplicates (Fig. 1). After screening 36,868 studies based on title and abstract, another 36,492 studies were excluded, leaving 67 studies for full-text assessment. Of these, 51 studies were further excluded as detailed in Fig. 1. Sixteen studies met the inclusion criteria and were included in the meta-analysis, providing data on 18,411 pregnant women. An additional search was conducted to identify published papers from 19 August 2023 to 28 May 2024. From the second search, we retrieved 4697 papers of which 1827 were duplicates. 2870 papers were screened based on the title and abstracts, and 2861 papers were excluded, full text review was performed on the nine 9 studies. Of these four studies met the inclusion criteria and were included in the meta-analysis (Fig. 1).

### Study characteristics

Five prospective cohort studies, seven cross-sectional studies, one case-control study, three cohort studies, one randomised control study and three retrospective studies were eligible for inclusion. The criteria used to diagnose MetS, and its components varied among the studies: seven studies used WHO criteria, two studies used the Chinese Medical Association criteria, seven studies used IDF criteria and ten studies used the NCEP-ATP III criteria: Supp. Table 1). The studies included in this review were from countries including Bosnia, Spain, Switzerland, Cameroon, Germany, Australia, Hungary, Sri Lanka, Brasil, Brazil, Iran Taiwan, Cuba, Angola, Serbia, USA and China. The age range of participants ranged from 18 to 50 years and the sample size ranged from 50 to 27,955. The time of assessment of MetS was between the first week and 30th weeks of pregnancy.

### Prevalence of MetS among pregnant women

**Fig. 2:**
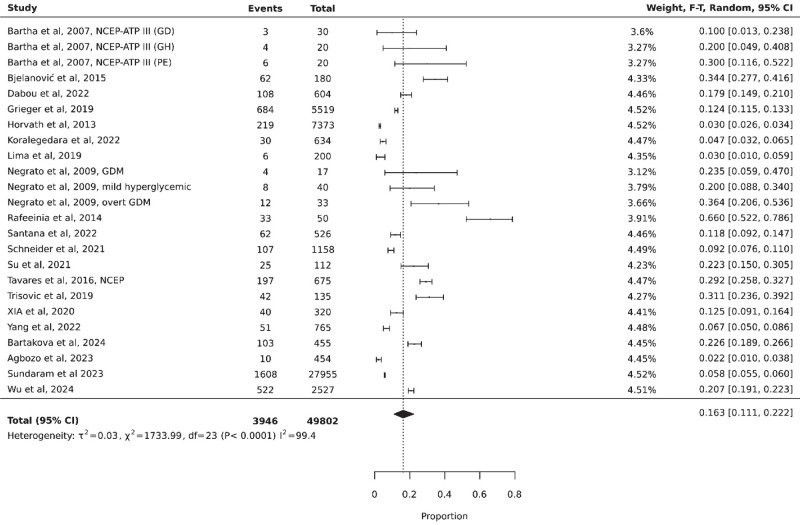
Prevalence of metabolic syndrome in pregnant women. NCEP National Cholesterol Education Program Adult Treatment Panel III, GDM Gestational Diabetes Mellitus

The prevalence of MetS in pregnancy was reported in 20 studies [12–31] which collectively included data from 49,802 participants. Among these, 3946 individuals were diagnosed with MetS. The pooled prevalence of MetS among pregnant women was 16.3% (95% CI 0.11–0.22, I^2^ = 99.4%; Fig. 2).

### Prevalence of MetS components

#### Obesity

**Fig. 3:**
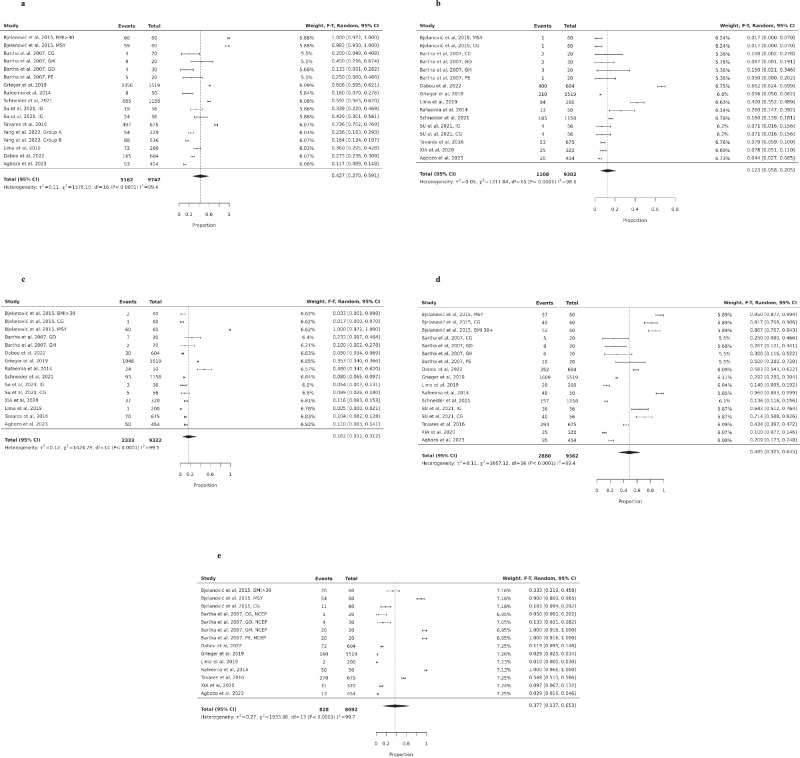
Prevalence of individual components of metabolic syndrome. **a** Prevalence of obesity among pregnant women. BMI body mass index, MSY metabolic syndrome, CG control group, GH gestational hypertension, GD gestational diabetes, PE preeclampsia. **b** Prevalence of low HDL among pregnant women. MSY metabolic syndrome, CG control group, GD gestational diabetes, GH gestational hypertension, PE preeclampsia, IG intervention group. **c** Prevalence of high fasting glucose among pregnant women. BMI body mass index, MSY metabolic syndrome, CG control group, GH gestational hypertension, GD gestational diabetes, IG intervention group, PE preeclampsia. **d** Prevalence of high triglycerides among pregnant women. MSY metabolic syndrome, CG control group, BMI body mass index, GD gestational diabetes, GH gestational hypertension, PE preeclampsia. **e** Prevalence of high blood pressure among pregnant women. BMI body mass index, MSY metabolic syndrome, CG control group, NCEP-ATP III National Cholesterol Education Program Adult Treatment Panel III

The prevalence of obesity was reported in ten studies, which collectively included data from 9.747 participants [12–15, 18, 20, 22–24, 27, 31]. Among these, 5162 participants were obese during pregnancy. The pooled prevalence of obesity among pregnant women was 42.7% (95% CI 0.27–0.59, I^2^ = 99.4%: Fig. 3a).

#### Low HDL cholesterol

The prevalence of low HDL cholesterol in pregnancy was reported in twelve studies, providing data from 9302 participants, of whom 1108 had low HDL [12–15, 18, 20, 22–24, 26, 31]. The pooled prevalence of low HDL cholesterol was 12.3% among pregnant women (95% CI 0.06–0.21, I^2^ = 98.6%; Fig. 3b).

#### High fasting glucose

Prevalence of high fasting glucose during pregnancy was reported in ten studies, providing data from 9322 participants, of whom 2333 had high fasting glucose [12–15, 18, 20, 22–24, 26, 31]. The pooled prevalence of high fasting glucose among pregnant women was 16.2% (95% CI 0.05–0.31, I^2^ = 99.5%; Fig. 3c).

#### High triglycerides

The prevalence of high triglycerides during pregnancy was reported in ten studies, providing data from 9362 participants, of whom 2880 had high triglycerides [12–15, 18, 20, 22–24, 26, 31]. The pooled prevalence of high triglycerides among pregnant participants was 48.5% (95% CI 0.33–0.65, I^2^ = 99.4%; Fig. 3d)

#### High blood pressure

The prevalence of high blood pressure was available in eight studies, providing data from 8092 participants, of whom 828 had high blood pressure [12–15, 18, 20, 24, 26, 31]. The pooled prevalence of high blood pressure among pregnant women was 37.7% (95% CI 0.27–0.65, I^2^ = 99.7%; Fig. 3e). All meta-analyses on prevalence of MetS components showed a high level of heterogeneity among studies.

### Prevalence of MetS based on the WHO, IDF and NCEP-ATP III diagnostic criteria

#### World Health Organisation criteria

Three studies used WHO criteria to diagnose MetS, providing data from 8078 participants, of whom 398 were diagnosed with MetS during pregnancy [12, 13, 16, 29]. The pooled prevalence of MetS during pregnancy according to the WHO criteria was 18.2% (95% CI 0.07–0.34, I^2^ = 97.7%; Supp. Fig. 1).

#### International diabetes federation criteria

Five studies used the IDF criteria to diagnose MetS, providing data from 7564 participants, of whom 959 were diagnosed with MetS during pregnancy [15, 22, 23, 26, 29]. The pooled prevalence of MetS during pregnancy according to the IDF criteria was 15% (95% CI 0.10–0.21, I^2^ = 95.5%; Supp. Fig. 2).

#### National cholesterol education program-adult treatment panel III criteria

Seven studies used the NCEP-ATP III criteria to diagnose MetS, providing data from 36,387 participants, of whom 2029 were diagnosed with MetS during pregnancy [12, 14, 16, 18, 20, 25, 30]. The pooled prevalence of MetS during pregnancy according to the NCEP-ATP III criteria was 17.2% (95% CI 0.07–0.32, I^2^ = 99.7%; Supp. Fig. 3).

### Prevalence of MetS based on gestational age at assessment

#### Prevalence of MetS prior to 16 weeks’ gestation

Eight studies reported on the prevalence of MetS prior to 16 weeks gestation. These studies included data from a total of 15,910 participants, of whom 1210 were diagnosed with MetS [13, 15–18, 21, 22, 26]. The prevalence of MetS prior to 16 weeks of gestation was 9.9% (95% CI 0.05–0.16, I^2^ = 99.2%; Supp. Fig. 4).

#### Prevalence of MetS at after 20 weeks’ gestation

Seven studies reported on the prevalence of MetS after 20 weeks’ gestation. These studies included data from 5483 participants, of whom 1118 participants were diagnosed with MetS [12, 14, 19, 20, 23–25, 27–29]. The prevalence of MetS after 20 week of gestation was 24.1% (95% CI 0.17–0.31, I^2^ = 96.3%; Supp. Fig. 5).

### Sensitivity analysis

Nine studies were identified as high-quality papers. Hence, the meta-analysis for the primary outcome was repeated with the inclusion of these high-quality papers and excluding the ten papers that were identified as moderate quality. These nine studies included data from 40,039 participants, of whom 2838 were diagnosed with MetS [12–15, 18, 24, 28–30]. The prevalence of MetS was 15.7% (95% CI 0.090–0.237, I^2^ = 99.5%; Supp. Fig. 6).

## Discussion

To the best of our knowledge, this is the first systematic review focusing on the prevalence of MetS and its components among pregnant women. Our results demonstrated that the overall pooled prevalence of MetS was 16.3%. The prevalence of MetS was 9.9% prior to 16 weeks gestation and increased to 24.1% after 20 weeks gestation. Additionally, the prevalence of MetS varied depending on the diagnostic criteria used.

Previous studies have shown that the rates of MetS among pregnant women varied throughout the world. The occurrence of MetS among pregnant women in the Asia-Pacific region, which includes the Philippines, China, Sri Lanka, Taiwan, Singapore, South Korea, Mongolia, and Malaysia, ranged from 11.9% to 37.1% while an Australian study reported a prevalence of 12.4% among nulliparous pregnant women. Studies from Europe have reported prevalence of MetS ranging from 2.2% to 22.6% among pregnant women. These findings suggest that ethnic diversity may contribute to the observed differences throughout the world.

Maternal metabolic adaptations to pregnancy may also contribute to the observed rates of MetS. During a normal pregnancy, total cholesterol, LDL and triglycerides start to increase after 20 weeks’ gestation. During the third trimester, maternal circulating lipid concentrations show a major increase, with triglycerides being elevated by approximately twofold, and total and LDL cholesterol by approximately 30–50% [32]. Another important metabolic adaptation is a change in insulin sensitivity. During early gestation, insulin sensitivity increases, promoting the uptake of glucose into adipose stores in anticipation of the increased energy demands of later pregnancy. However, as pregnancy progresses, a surge in maternal and placental hormones, including oestrogen, progesterone, leptin, cortisol, placental lactogen, and placental growth hormone collectively promote a state of insulin resistance [32]. Additionally, women typically gain approximately 4.9–9 kg throughout pregnancy [33]. These physiological changes are required to meet the needs of the growing foetus. Our finding of higher prevalence of MetS during the latter part of pregnancy may be attributed to these normal physiological changes.

Our results also showed that prevalence of MetS differed depending on the diagnostic criteria used. When stratified according to the diagnostic criteria, the most common occurrence rate of 19.3% was observed when the NCEP-ATP III criteria were applied. These criteria require the presence of abdominal obesity, as well as two of five other risk factors. The global prevalence of obesity is increasing rapidly, with approximately 45.1% of pregnant women found to be overweight or obese [1]. Women typically gain 4.9–9 kg weight throughout pregnancy, with most weight gain occurring during the latter part of pregnancy. This high prevalence of obesity likely contributes to the elevated rates of MetS when using the NCEP-ATP III criteria.

Our meta-analysis showed that MetS is detected in approximately one-fifth of pregnant women. Normal pregnancy is characterised by a pro-inflammatory, pro-thrombotic state with high insulin resistance and hyperlipidaemia, both of which are typically considered in the diagnosis of MetS. Insulin resistance promotes inflammation and impairs the function of the blood vessels, contributing to increased cardiovascular risks. Hyperlipidaemia contributes to the development of atherosclerosis, a key factor in cardiovascular disease [34]. Previous research has shown a consistent association between major pregnancy complications and MetS. A previous study of over 5000 pregnant women reported that more than half of women who had MetS in early pregnancy developed a major pregnancy complication including preeclampsia or gestational diabetes [15]. Approximately 36% of women who experience a major pregnancy complication are also diagnosed with MetS by six months postpartum [35]. At present, it is not clear whether developing a major pregnancy complication including preeclampsia or gestational diabetes increases the risk of subsequent metabolic syndrome and coronary artery disease or whether women with existing cardio-metabolic risk factors develop major pregnancy complications and subsequently coronary artery disease. A previous study has shown that women who have one or more of the components of MetS in early pregnancy are approximately six times more likely to be diagnosed with MetS at 10 years postpartum than women who do not have MetS components in early pregnancy [36] suggesting that screening for MetS and its components in early pregnancy may help to identify women at risk of CVD later in life. Early detection and management of these risk factors may help optimise maternal and foetal outcomes during pregnancy.

We acknowledge some limitations in our systematic review. First, a high level of heterogeneity was detected in all the outcomes. Subgroup analyses stratified by gestational age at assessment and diagnostic criteria failed to reduce the heterogeneity. Sensitivity analysis based on study quality also failed to reduce the heterogeneity. These findings indicate that there are other differences across the included studies which may include genetic and ethnic diversity. Second, we limited our search to articles that were published in English only and may have missed important studies that were published in other languages. Large prospective cohort studies with MetS assessments in early pregnancy will help better understand the prevalence of MetS among pregnant women.

## Conclusion

This review demonstrates that MetS is detected in approximately one fifth of pregnant women. The differences in prevalence based on the diagnostic criteria and gestational age at assessment suggests that pregnancy-specific diagnostic criteria are required to better understand the true prevalence of MetS among pregnant women. However, an overall 16% prevalence of MetS among pregnant women suggests that screening in early pregnancy may identify young women at risk of future coronary artery disease who will benefit by early preventive measures.

## Supplementary information

MOOSE_Guidliness _Checklist

Supplementary appendix

## References

[1] S. Mendis, I. Graham, J. Narula, Addressing the global burden of cardiovascular diseases; need for scalable and sustainable frame-works. Global Heart. **17**, 48 (2022) 36051329 10.5334/gh.1139 PMC9336686

[2] M. Vaduganathan, G.A. Mensah, J.V. Turco, V. Fuster, G.A. Roth, The global burden of cardiovascular diseases and risk: a compass for future health. *J. Am. Coll. Cardiol.* **80**, 2361–2371 (2022) 10.1016/j.jacc.2022.11.005 36368511

[3] P.L. Huang, A comprehensive definition for metabolic syndrome. Dis. Model Mech. **2**, 231–237 (2009) 19407331 10.1242/dmm.001180 PMC2675814

[4] S. Mottillo, K.B. Filion, J. Genest, L. Joseph, L. Pilote, P. Poirier et al. The metabolic syndrome and cardiovascular risk: a systematic review and meta-analysis. J. Am. Coll. Cardiol. **56**(14), 1113–1132 (2010) 20863953 10.1016/j.jacc.2010.05.034

[5] J.J. Noubiap, J.R. Nansseu, E. Lontchi-Yimagou, J.R. Nkeck, U.F. Nyaga, A.T. Ngouo et al. Geographic distribution of metabolic syndrome and its components in the general adult population: a meta-analysis of global data from 28 million individuals. Diab. Res. Clin. Pract. **188**, 109924 (2022) 10.1016/j.diabres.2022.109924 35584716

[6] E. Kassi, P. Pervanidou, G. Kaltsas, G. Chrousos, Metabolic syndrome: definitions and controversies. BMC Med. **5**, 9 (2011) 10.1186/1741-7015-9-48 PMC3115896 21542944

[7] P. Zimmet, D. Magliano, Y. Matsuzawa, G. Alberti, J. Shaw, The metabolic syndrome: a global public health problem and a new definition. J. Atheroscler. Thromb. **12**, 295–300 (2005) 16394610 10.5551/jat.12.295

[8] K.G.M.M. Alberti, R.H. Eckel, S.M. Grundy, P.Z. Zimmet, J.I. Cleeman, K.A. Donato et al. Harmonizing the metabolic syndrome: a joint interim statement of the International Diabetes Federation Task Force on Epidemiology and Prevention; National Heart, Lung, and Blood Institute; American Heart Association; World Heart Federation; International Atherosclerosis Society; and International Association for the Study of Obesity. Circulation **120**, 1640–1645 (2009) 19805654 10.1161/CIRCULATIONAHA.109.192644

[9] M. Sanghavi, J.D. Rutherford, Cardiovascular physiology of pregnancy. Circulation **130**(12), 1003–1008 (2014) 25223771 10.1161/CIRCULATIONAHA.114.009029

[10] B.S. Brooke, T.A. Schwartz, T.M. Pawlik, MOOSE reporting guidelines for meta-analyses of observational studies. JAMA Surg. **156**(8), 787–788 (2021) 33825847 10.1001/jamasurg.2021.0522

[11] M. Zachary, A. Edoardo, T. Catalin, S. Cindy, P. Kylie, F. James, L. Craig, S. Mathew, M. Sandeep, L. Lucylynn, M. Alexandra, P. Micah, P. Alan, J. Zoe, The development of software to support multiple systematic review types: the Joanna Briggs institute system for the unified management, assessment and review of information (JBI SUMARI). Int. J. Evid.-Based Health **17**(1), 36–43 (2019) 10.1097/XEB.0000000000000152 30239357

[12] J.L. Bartha, F. González-Bugatto, R. Fernández-Macías, N.L. González-González, R. Comino-Delgado, B. Hervías-Vivancos, Metabolic syndrome in normal and complicated pregnancies. Eur. J. Obstet. Gynecol. Reprod. Biol. **137**(2), 178–184 (2008) 17681419 10.1016/j.ejogrb.2007.06.011

[13] V. Bjelanović, D. Babić, D. Hodžić, A. Bjelanović, T. Krešić, A. Dugandžić-Šimić et al. Correlation of psychological symptoms with cortisol and CRP levels in pregnant women with metabolic syndrome. Psychiatr. Danub **27**, 578–585 (2015) 26657985

[14] S. Dabou, N.S. Ongbayokolak, L.F. Sama, E.M. Foking, N.M. Kamdom, P.B. Telefo, Metabolic syndrome during pregnancy: prevalence and determinants among pregnant women followed-up at the Dschang district hospital, west region of Cameroon. Diab. Metab. Syndr. Obes. **15**, 743–753 (2022) 10.2147/DMSO.S348040 PMC8906707 35280500

[15] J.A. Grieger, L.E. Grzeskowiak, L.G. Smithers, T. Bianco-Miotto, S.Y. Leemaqz, P. Andraweera et al. Metabolic syndrome and time to pregnancy: a retrospective study of nulliparous women. BJOG **126**(7), 852–862 (2019) 30734474 10.1111/1471-0528.15647

[16] B. Horvath, T. Bodecs, I. Boncz, J. Bodis, Metabolic syndrome in normal and complicated pregnancies. Metab. Syndr. Relat. Disord. **11**(3), 185–188 (2013) 23438156 10.1089/met.2012.0086

[17] I.S. Koralegedara, J. Warnasekara, I. Jayasinghe, T. Agampodi, S. Agampodi, K. Gedara Dayaratne. Non-alcoholic fatty liver disease among pregnant women with metabolic syndrome: should nutri-tional intervention be a priority? A cross-sectional study in rural Sri Lanka. Curr. Dev. Nutr **6**, 680 (2022)

[18] M. Lima, A.S.O. Melo, A.S.S. Sena, V. de Oliveira Barros, M.M.R. Amorim, Metabolic syndrome in pregnancy and postpartum: prevalence and associated factors. Rev. Assoc. Med Bras. **65**(12), 1489–1495 (2019) 31994631 10.1590/1806-9282.65.12.1489

[19] C.A. Negrato, L. Jovanovic, A. Rafacho, M.A. Tambascia, B. Geloneze, A. Dias et al. Association between different levels of dysglycemia and metabolic syndrome in pregnancy. Diabetol. Metab. Syndr. **1**(1), 3 (2009) 19825195 10.1186/1758-5996-1-3 PMC2758580

[20] A. Rafeeinia, A. Tabandeh, S. Khajeniazi, A. Marjani, Metabolic syndrome in preeclampsia women in Gorgan. Open Biochem J. **8**, 94–99 (2014) 25553139 10.2174/1874091X01408010094 PMC4279033

[21] A. Artiles-Santana, N.L. Sarasa-Muñoz, E. Izaguirre-Castellanos, E.Á. Guerra-González, O. Cañizares-Luna, Validation of a new diagnostic index to determine metabolic obesity phenotypes in normal-weight women in early pregnancy. MEDICC Rev. **24**, 30–35 (2022) 36417332 10.37757/MR2022.V24.N3-4.3

[22] A.K. Schneider, S.Y. Leemaqz, J. Dalton, P.E. Verburg, B.W. Mol, G.A. Dekker et al. The interaction between metabolic syndrome and physical activity, and risk for gestational diabetes mellitus. Acta Diabetol. **58**(7), 939–947 (2021) 33743081 10.1007/s00592-021-01696-9

[23] M.C. Su, A.S. Chao, M.Y. Chang, Y.L. Chang, Chien, L. Chen et al. Effectiveness of a nurse-led web-based health management in preventing women with gestational diabetes from developing metabolic syndrome. J. Nurs. Res. **29**, e176 (2021) 34570053 10.1097/jnr.0000000000000456

[24] H. Dos Prazeres Tavares, D.C.D.M. Dos Santos, J.F. Abbade, C.A. Negrato, P.A. De Campos, I.M.P. Calderon et al. Prevalence of metabolic syndrome in non-diabetic, pregnant Angolan women according to four diagnostic criteria and its effects on adverse perinatal outcomes. Diabetol. Metab. Syndr. **8**, 27 (2016) 27006707 10.1186/s13098-016-0139-3 PMC4802648

[25] M. Trisovic, O. Mladenovic, J. Bila, K. Lalić, D. Kisic Tepavcevic, The predictive value of metabolic syndrome in the evaluation of pregnancy course and outcome. Clin. Exp. Obstet. Gynecol. **46**(5), 776–778 (2019)

[26] B. Xia, W. Wang, Y. Lu, C. Chen, Helicobacter pylori infection increases the risk of metabolic syndrome in pregnancy: a cohort study. Ann. Transl. Med. **8**(14), 875–875 (2020) 32793719 10.21037/atm-20-4863 PMC7396788

[27] X. Yang, R. Jiang, X. Yin, G. Wang, Pre-BMI and lipid profiles in association with the metabolic syndrome in pregnancy with advanced maternal age. Contrast Media Mol. Imaging **2022**, 4332006 (2022) 35854775 10.1155/2022/4332006 PMC9288333

[28] H. Wu, M.H. Yi, B.G. Liu et al. Association of gestational metabolic syndrome with the Chinese Healthy Eating Index in mid-pregnancy: a cross-sectional study. Nutr. Metab. **21**, 8 (2024) 10.1186/s12986-024-00780-5 PMC10811910 38279139

[29] V. Bartakova, K. Chalasova, L. Pacal, V. Tapalova, J. Machal, P. Janku, K. Kankova, Metabolic syndrome prevalence in women with gestational diabetes mellitus in the second trimester of gravidity. J. Clin. Med. **13**, 1260 (2024) 38592122 10.3390/jcm13051260 PMC10932344

[30] V.L. Sundaram, R. Lamichhane, A. Cecchetti, S. Arthur, U. Murughiyan, Maternal and neonatal outcomes in women with metabolic syndrome and substance use disorder. Life **13**, 1933 (2023) 37763336 10.3390/life13091933 PMC10533184

[31] F. Agbozo, J. Amenu, A. Abubakari, A. Jahn, Dyslipidemia, hyperglycemia, hypertension and obesity as biomarkers of metabolic syndrome among pregnant women in non-urbanized Ghana. Ann. Nutr. Metab. **79**, 614–615 (2023)

[32] J.W.C.M. Mulder, D.M. Kusters, J.E. Roeters van Lennep, B.A. Hutten, Lipid metabolism during pregnancy: consequences for mother and child. Curr. Opin. Lipido. **35**(3), 133–140 (2024) 10.1097/MOL.0000000000000927 PMC11064913 38408036

[33] U. Moll, H. Olsson, M. Landin-Olsson, Impact of pregestational weight and weight gain during pregnancy on long-term risk for diseases. PLoS One **12**(1), e0168543 (2017) 28045917 10.1371/journal.pone.0168543 PMC5207749

[34] G. Girardi, A.A. Bremer, The intersection of maternal metabolic syndrome, adverse pregnancy outcomes, and future metabolic health for the mother and offspring. Metab. Syndrome Relat. Disord. **20**, 251–254 (2022) 10.1089/met.2021.0124 PMC9360170 35384734

[35] E. Aldridge, M. Pathirana, M. Wittwer, S. Sierp, S.Y. Leemaqz, C.T. Roberts et al. Prevalence of metabolic syndrome in women after maternal complications of pregnancy: an observational cohort analysis. Front. Cardiovasc. Med. **14**, 9 (2022) 10.3389/fcvm.2022.853851 PMC8963931 35360031

[36] P.H. Andraweera, M.D. Plummer, A. Garrett, S. Leemaqz, M.R. Wittwer, E. Aldridge, M.M. Pathirana, G.A. Dekker, C.T. Roberts, M.A. Arstall, Early pregnancy cardio metabolic risk factors and the prevalence of metabolic syndrome 10 years after the first pregnancy. PLoS One **18**(1), e0280451 (2023) 36662760 10.1371/journal.pone.0280451 PMC9858479

