# Supplementary material for: Prevalence of metabolic syndrome among pregnant women: a systematic review and meta-analysis
Source: Endocrine. 2025 Jan 22;88(2):398–409. doi: 10.1007/s12020-025-04160-8 (PMC12069128; doi:10.1007/s12020-025-04160-8)
Supplement: Supplementary file 2 — Supplementary appendix [file 12020_2025_4160_MOESM2_ESM.docx]

**Supplement A: Search Strategy prepared for Pubmed, Embase, Web of science, Scopus and CINHL.**

1# "metabolic syndrome"[mh] OR "Triglycerides"[mh:noexp] OR "Lipoproteins, HDL"[mh] OR "blood pressure"[mh:noexp] OR "body mass index"[mh] OR "Lipoproteins, LDL"[mh] OR metabolic syndrome[tiab] OR triglycerides[tiab] OR high density lipoprotein[tiab] OR HDL[tiab] OR BMI[tiab] OR body mass index[tiab] OR low density lipoprotein[tiab] OR LDL[tiab] OR Diastolic Pressure[tiab] OR diastolic blood pressure[tiab] OR systolic pressure[tiab] OR systolic blood pressure[tiab] OR fasting plasma glucose[tiab] OR fasting glucose level[tiab] OR central obesity[tiab] OR abdominal obesity[tiab] OR waist circumference[tiab] OR hypertension[tiab] OR high blood pressure[tiab] OR hypertriglyceridemia[tiab] OR maternal obesity[tiab]

2# "prevalence"[mh] OR prevalence[tiab] OR occurrence[tiab] OR incidence[tiab]

3# "pregnancy"[mh] OR pregnan*[tiab] OR gestation[tiab] OR maternal[tiab]

4# OR 3# 1# 2#

**Supp Fig. 1** Prevalence of MetS among pregnant women according to the WHO criteria


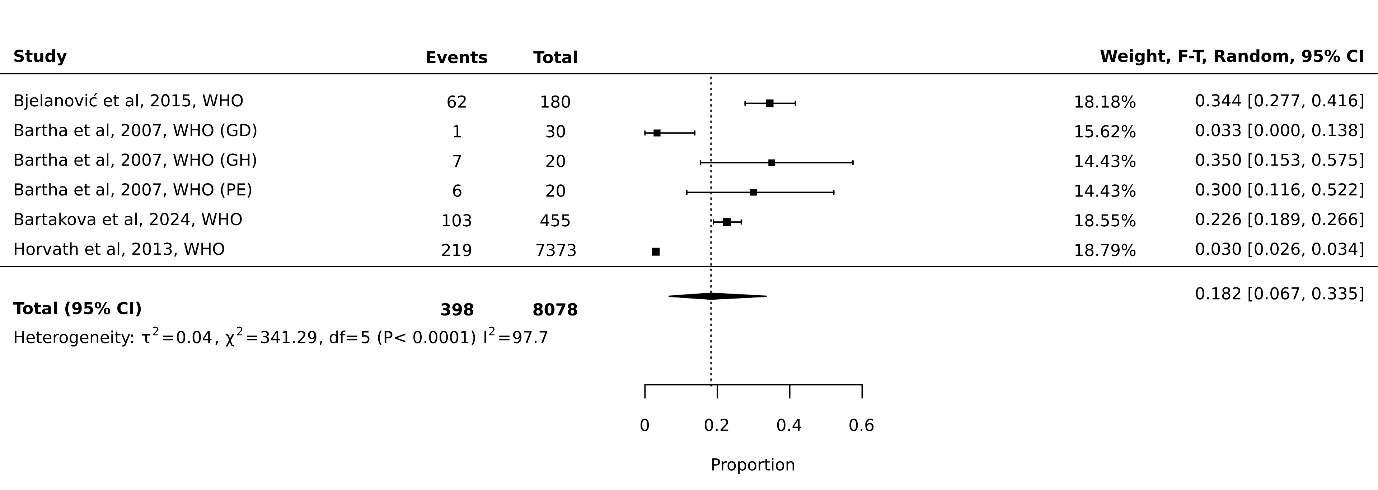


WHO, World Health Organisation; GD, gestational diabetes; GH, gestational hypertension; PE, preeclampsia.

**Supp Fig. 2** Prevalence of MetS among pregnant women according to the IDF criteria


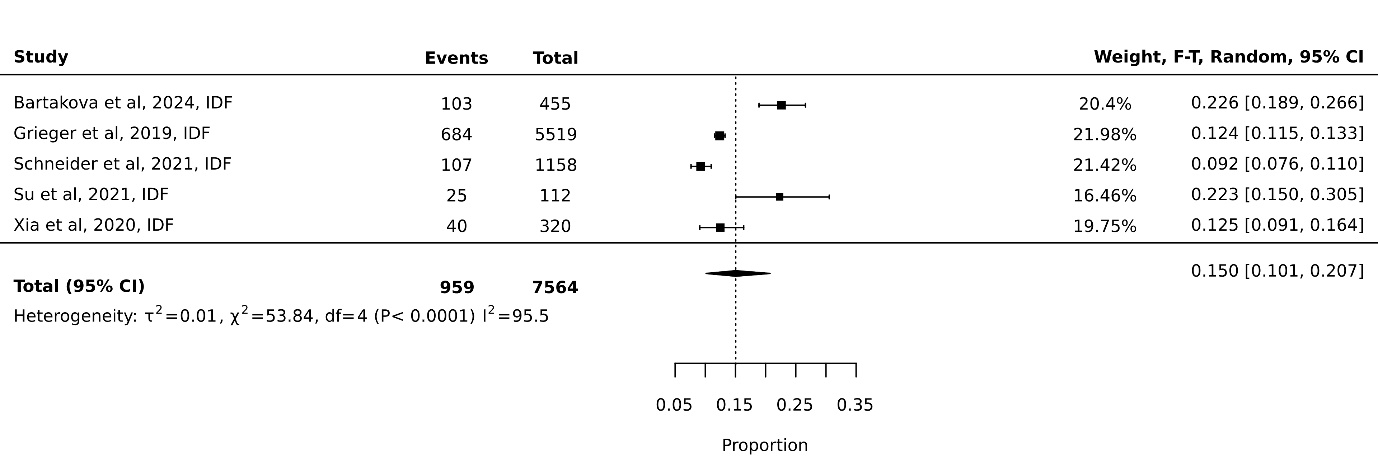


IDF, International Diabetes Federation.

**Supp Fig. 3** Prevalence of MetS among pregnant women according to the NCEP-ATP III criteria


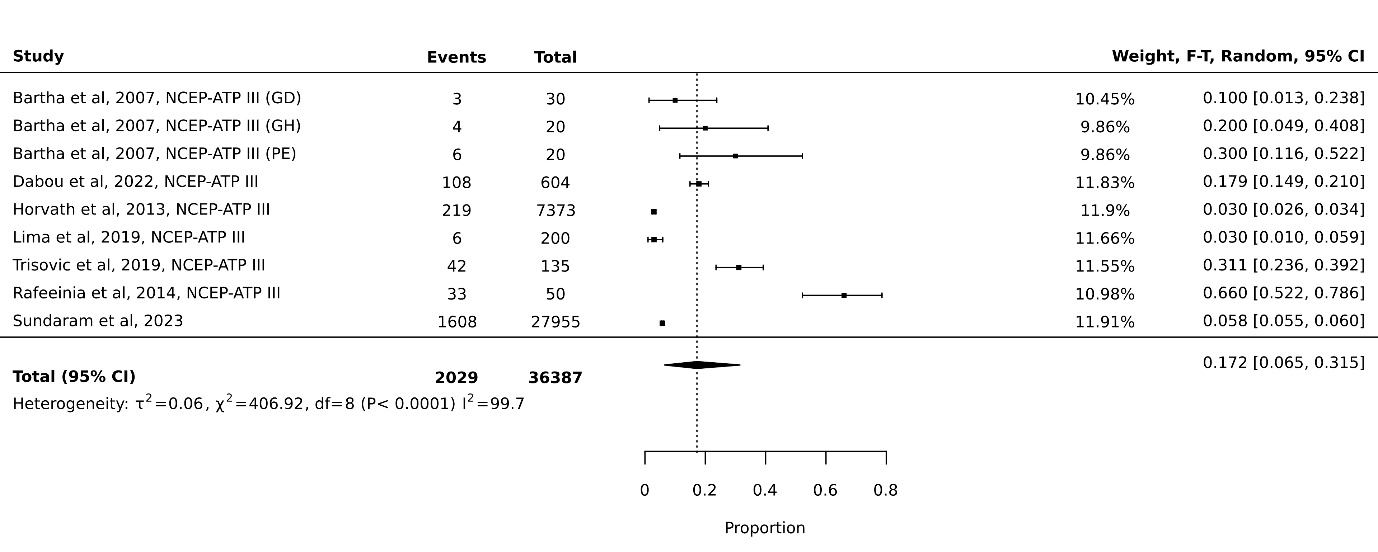


NCEP-ATP III,; GD, Gestational Diabetes; GH, gestational hypertension; PE, preeclampsia.

**Supp Fig. 4** Prevalence of MetS among pregnant women at <16 weeks gestation


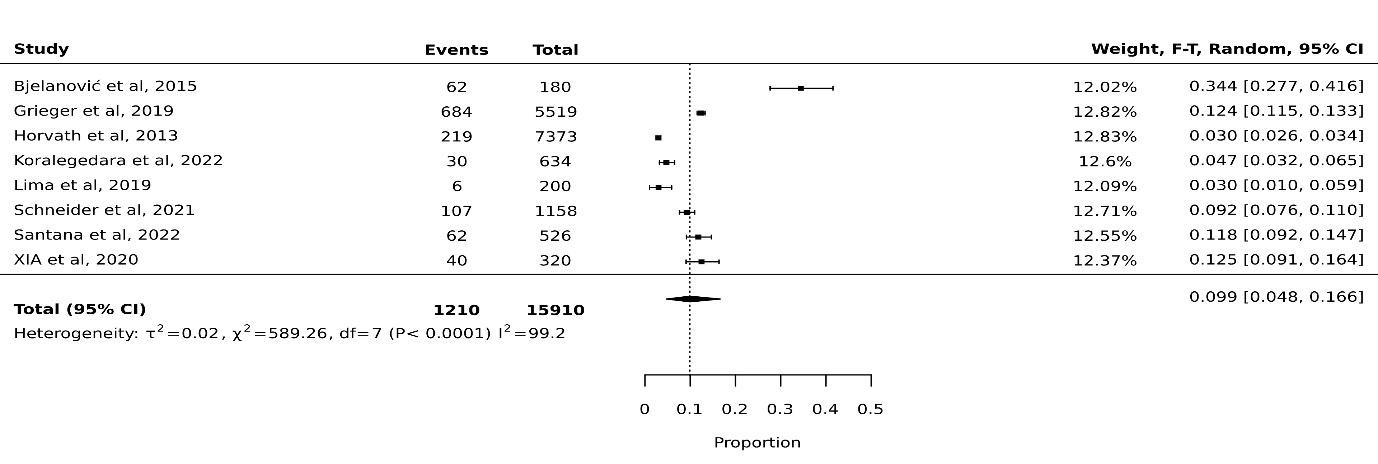


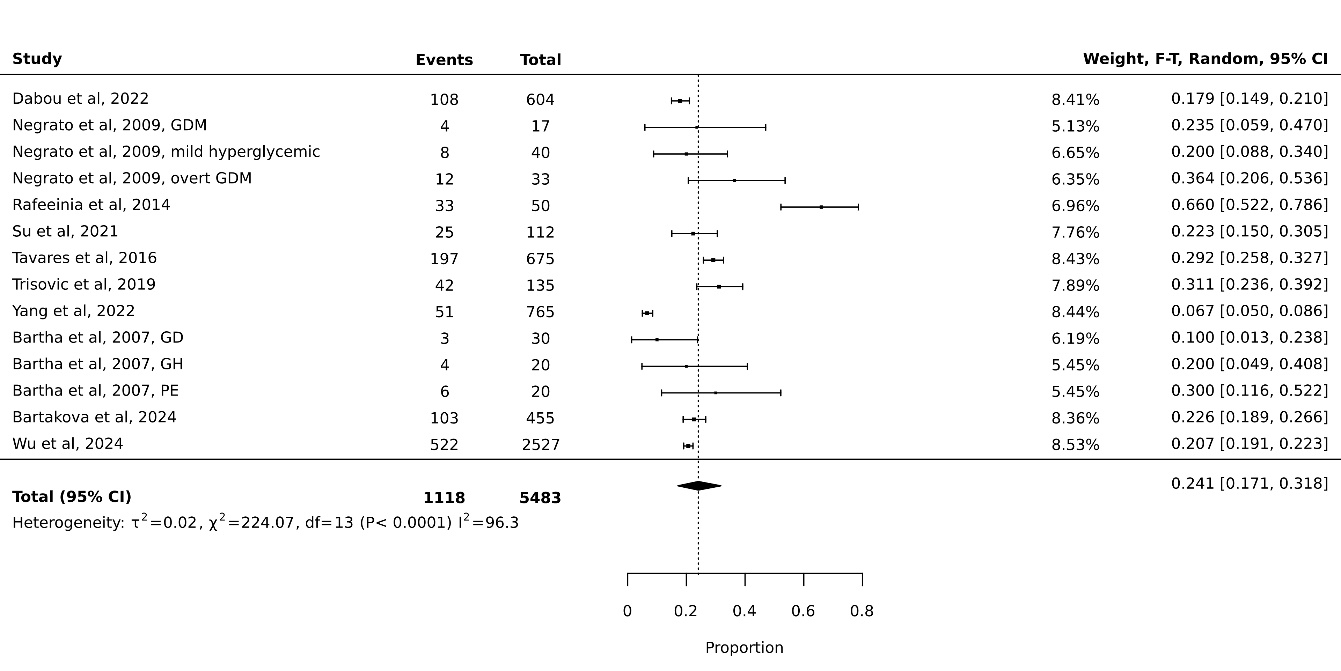
**Supp. Fig. 5** Prevalence of MetS among pregnant women at >20 weeks gestation

GDM, gestational diabetes Mellitus; GH, gestational hypertension; PE, preeclampsia.

**Supp. Fig. 6**: Sensitivity analysis of high quality studies.


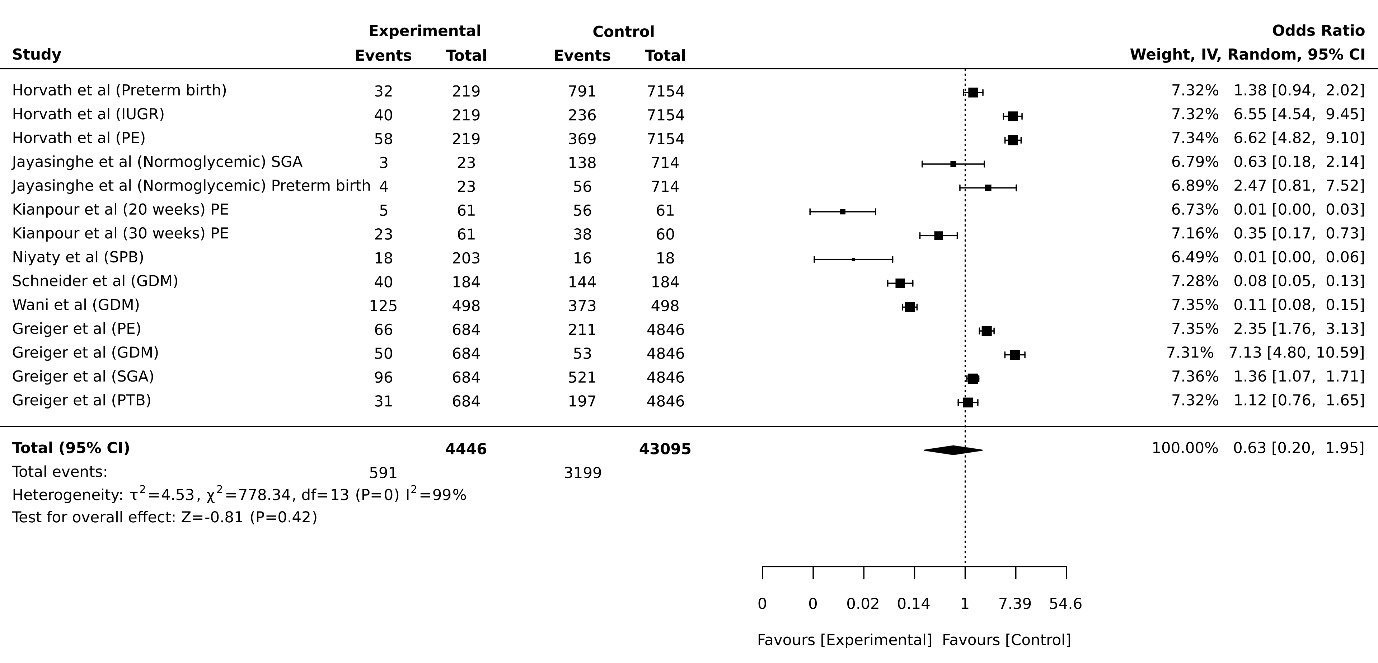


**Supp. Table 2** Quality Assessment (JBI Sumari critical appraisal checklist)

|  | **1** | **2** | **3** | **4** | **5** | **6** | **7** | **8** | **9** | **Overall score** |
| --- | --- | --- | --- | --- | --- | --- | --- | --- | --- | --- |
|  | **Sample Frame** | **Study participants** | **Sample size** | **Study subjects** | **Data analysis** | **Valid methods** | **Conditions measured** | **Appropriate statistical analysis** | **Response rate** | **Score** |
| Agbozo et al, [2023] | Yes | Yes | Yes | Yes | No | Yes | Yes | No | No | 6 |
| Bartakova et al, [2024] | Yes | Yes | Yes | Yes | Yes | Yes | Yes | Yes | Yes | 9 |
| Bjelanović et al. [2015] | Yes | Yes | No | Yes | Yes | Yes | Yes | Yes | Yes | 8 |
| Bartha et al. [2007] | Yes | Yes | No | Yes | Yes | Yes | Yes | Yes | Yes | 8 |
| Dabou et al. [2022] | Yes | Yes | Yes | Yes | Yes | Yes | Yes | Yes | Yes | 9 |
| Grieger et al. [2019] | Yes | No | No | Yes | Yes | Yes | Yes | Yes | Yes | 7 |
| Horvath et al. [2013] | Yes | Yes | No | Yes | Yes | Yes | Yes | Yes | Yes | 8 |
| Koralegedara et al. [2022] | Yes | Yes | Yes | Yes | No | Yes | Yes | No | No | 6 |
| Lima et al. [2019] | Yes | Yes | Yes | Yes | Yes | Yes | Yes | Yes | Yes | 9 |
| Negrato et al. [2009] | No | No | No | Yes | Yes | Yes | Yes | Yes | Yes | 6 |
| Rafeeinia et al. [2014] | No | Yes | No | No | No | Yes | Yes | Yes | Yes | 5 |
| Schneider et al. [2021] | Yes | Yes | No | Yes | No | Yes | Yes | Yes | No | 6 |
| SU et al. [2021] | Yes | Yes | Yes | Yes | No | Yes | Yes | Yes | No | 7 |
| Santana et al. [2022] | Yes | Yes | No | Yes | No | Yes | Yes | Yes | No | 6 |
| Sundaram et al, [2023] | Yes | Yes | Yes | Yes | No | Yes | Yes | Yes | Yes | 8 |
| Tavares et al. [2016] | Yes | Yes | No | Yes | Yes | Yes | Yes | Yes | Yes | 8 |
| Trisovic et al. [2019] | Yes | Yes | No | Yes | No | Yes | Yes | Yes | Yes | 7 |
| Wu et al, [2024] | Yes | Yes | Yes | Yes | Yes | Yes | Yes | Yes | Yes | 9 |
| Xia et al. [2020] | Yes | Yes | No | No | Yes | Yes | Yes | Yes | Yes | 7 |
| Yang et al. [2022] | Yes | Yes | No | Yes | No | Yes | Yes | Yes | Yes | 7 |

Low quality: 1-4, Moderate quality: 5-7, High quality: 8-10
